# Supplementary material for: Quantitative analysis of transcriptome dynamics provides novel insights into developmental state transitions
Source: BMC Genomics. 2022 Oct 23;23:723. doi: 10.1186/s12864-022-08953-3 (PMC9588240; doi:10.1186/s12864-022-08953-3)
Supplement: Supplementary file 1 — Additional file 1: Supplemental Figure 1. Xenopus blastula explants can be reprogrammed to adopt any lineage. (A-E) RNA Seq TPM expression over time of (A) average of all monotonically decreasing genes with a >25fold decrease between stage 9 and 13 and maximum TPM of 20 at stage 13, (B) epidermal marker Dlx3, (C) neural marker Otx2, (D) mesoderm marker Evx1 (E) endoderm marker Endodermin. Graphs are sums of S+L allele. Width of lines represents SEM of three biological replicates. [file 12864_2022_8953_MOESM1_ESM.pdf]

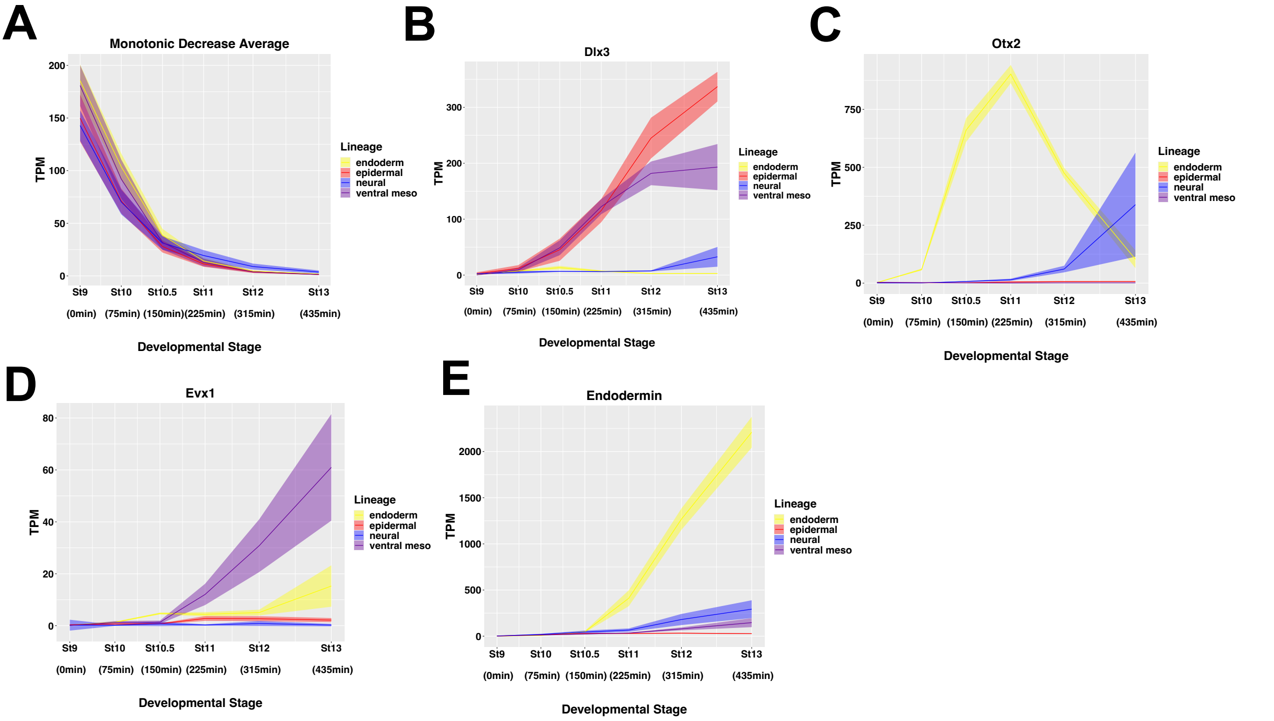

Supplemental Figure 1. Xenopus blastula explants can be reprogrammed to adopt any lineage. (A-E) RNA Seq TPM expression over time of (A) average of all monotonically decreasing genes with a >25fold decrease between stage 9 and 13 and maximum TPM of 20 at stage 13, (B) epidermal marker *Dlx3*, (C) neural marker *Otx2,* (D) mesoderm marker *Evx1* (E) endoderm marker *Endodermin*. Graphs are sums of S+L allele. Width of lines represents SEM of three biological replicates.
