# Supplementary material for: Quantitative analysis of transcriptome dynamics provides novel insights into developmental state transitions
Source: BMC Genomics. 2022 Oct 23;23:723. doi: 10.1186/s12864-022-08953-3 (PMC9588240; doi:10.1186/s12864-022-08953-3)
Supplement: Supplementary file 2 — Additional file 2: Supplemental Figure 2. Transcription Factor UpSet Plots and Zygotic PCA. (A-E) UpSet plots of transcription factors expressed at a minimum of 10 TPM at (A) stage 10, (B) stage 10.5, (C) stage 11, (D) stage 12, (E) stage 13. X-axes show genes unique to each lineage and overlapping in all different combinations of lineages ordered from largest number of genes to smallest. (F) PCA on only zygotically expressed genes [file 12864_2022_8953_MOESM2_ESM.pdf]

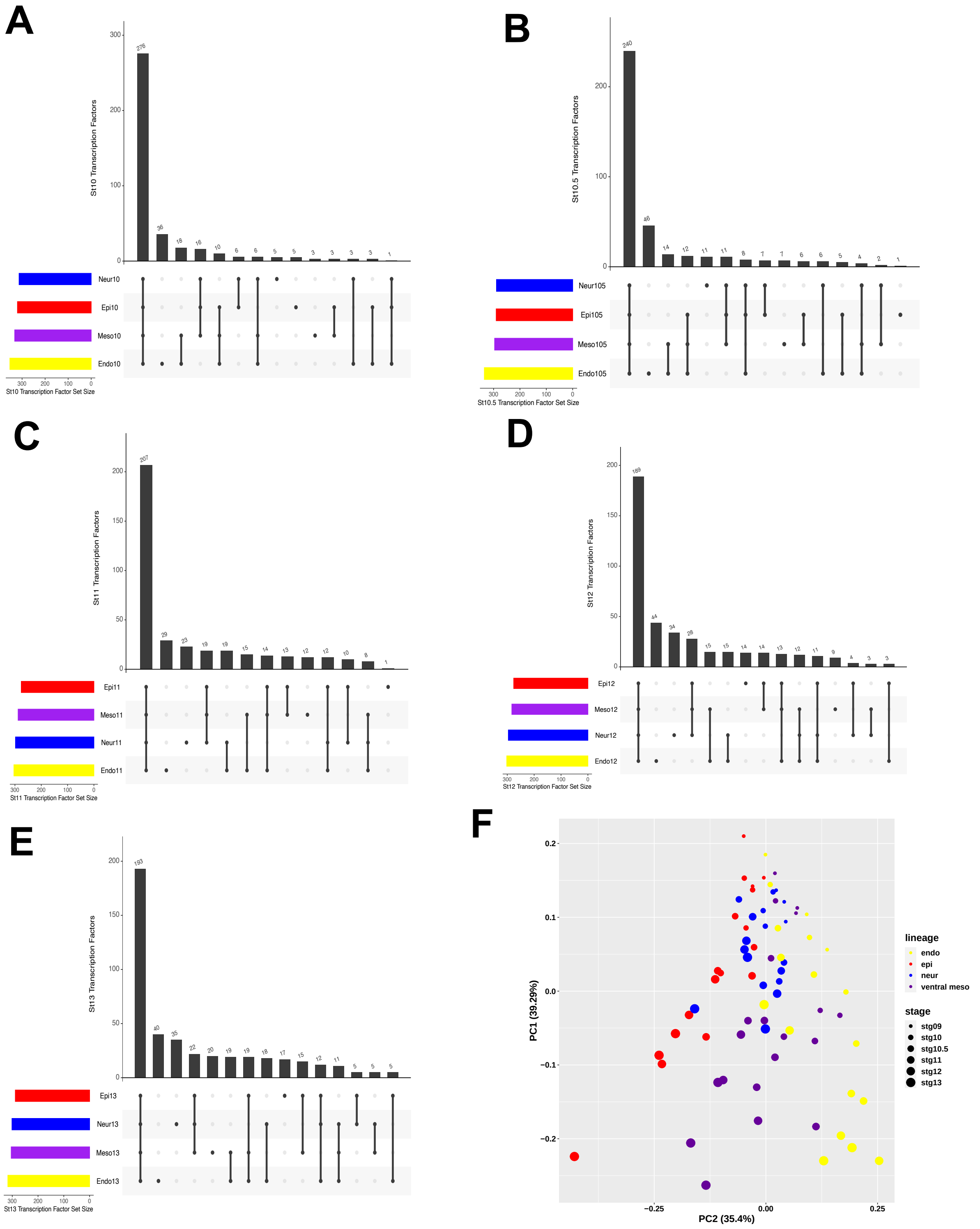

Supplemental Figure 2. Transcription Factor UpSet Plots and Zygotic PCA. (A-E) UpSet plots of transcription factors expressed at a minimum of 10 TPM at (A) stage 10, (B) stage 10.5, (C) stage 11, (D) stage 12, (E) stage 13. X-axes show genes unique to each lineage and overlapping in all different combinations of lineages ordered from largest number of genes to smallest. (F) PCA on only zygotically expressed genes.
