# Supplementary material for: Quantitative analysis of transcriptome dynamics provides novel insights into developmental state transitions
Source: BMC Genomics. 2022 Oct 23;23:723. doi: 10.1186/s12864-022-08953-3 (PMC9588240; doi:10.1186/s12864-022-08953-3)
Supplement: Supplementary file 3 — Additional file 3: Supplemental Figure 3. Temporal DESeq UpSet Plots. (A-J) UpSet plots of Differentially Expressed Genes (A) increased between stages 9 and 10, (B) decreased between stages 9 and 10, (C) increased between stages 10 and 10.5, (D) decreased between stages 10 and 10.5, (E) increased between stages 10.5 and 11, (F) decreased between stages 10.5 and 11, (G) increased between stages 11 and 12, (H) decreased between stages 11 and 12, (I) increased between stages 12 and 13, (J) decreased between stages 12 and 13. X-axes show genes unique to each lineage and overlapping in all different combinations of lineages ordered from largest number of genes to smallest. [file 12864_2022_8953_MOESM3_ESM.pdf]

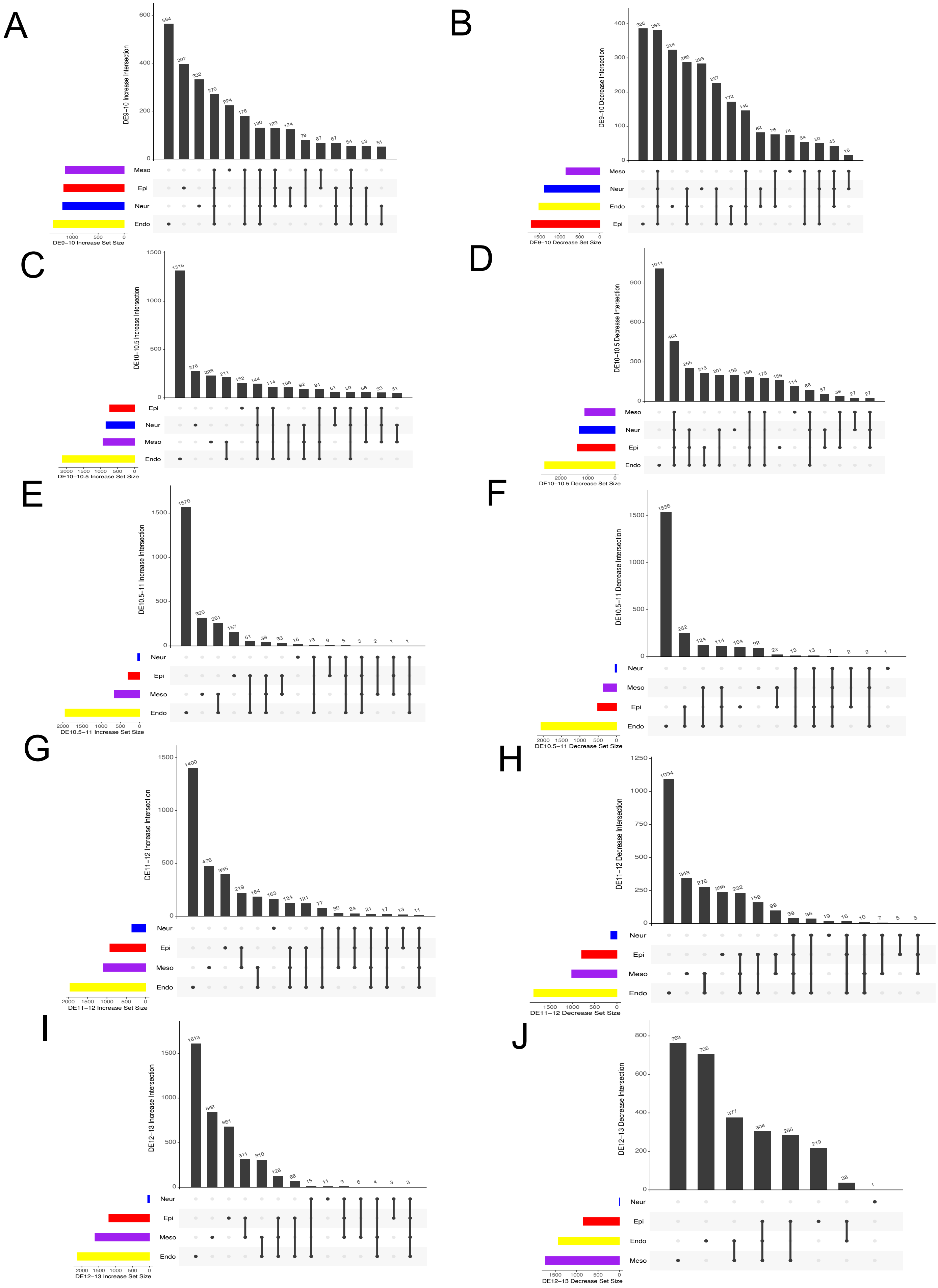

Supplemental Figure 3. Temporal DESeq UpSet Plots. (A-J) UpSet plots of Differentially Expressed Genes (A) increased between stages 9 and 10, (B) decreased between stages 9 and 10, (C) increased between stages 10 and 10.5, (D) decreased between stages 10 and 10.5, (E) increased between stages 10.5 and 11, (F) decreased between stages 10.5 and 11, (G) increased between stages 11 and 12, (H) decreased between stages 11 and 12, (I) increased between stages 12 and 13, (J) decreased between stages 12 and 13. X-axes show genes unique to each lineage and overlapping in all different combinations of lineages ordered from largest number of genes to smallest.
