# Supplementary material for: Quantitative analysis of transcriptome dynamics provides novel insights into developmental state transitions
Source: BMC Genomics. 2022 Oct 23;23:723. doi: 10.1186/s12864-022-08953-3 (PMC9588240; doi:10.1186/s12864-022-08953-3)
Supplement: Supplementary file 4 — Additional file 4: Supplemental Figure 4. Epidermal and Neural Lineages Diverge at St 10.5 (A) Number of enriched Neural GO Terms in genes significantly higher in the neural lineage (blue) and epidermal lineage (red) at each developmental stage. (B) Heatmap of the top 20 DE genes by Log2FC with a minimum expression of 10TPM between the epidermal and neural lineages at stage 10.5 (C) KEGG enrichment analysis of genes differentially expressed between epidermal and neural lineage at each developmental stage. Genes significantly increased in the epidermal lineage are enriched for TGF-beta genes, as defined by KEGG database from stages 10.5-13. [file 12864_2022_8953_MOESM4_ESM.pdf]

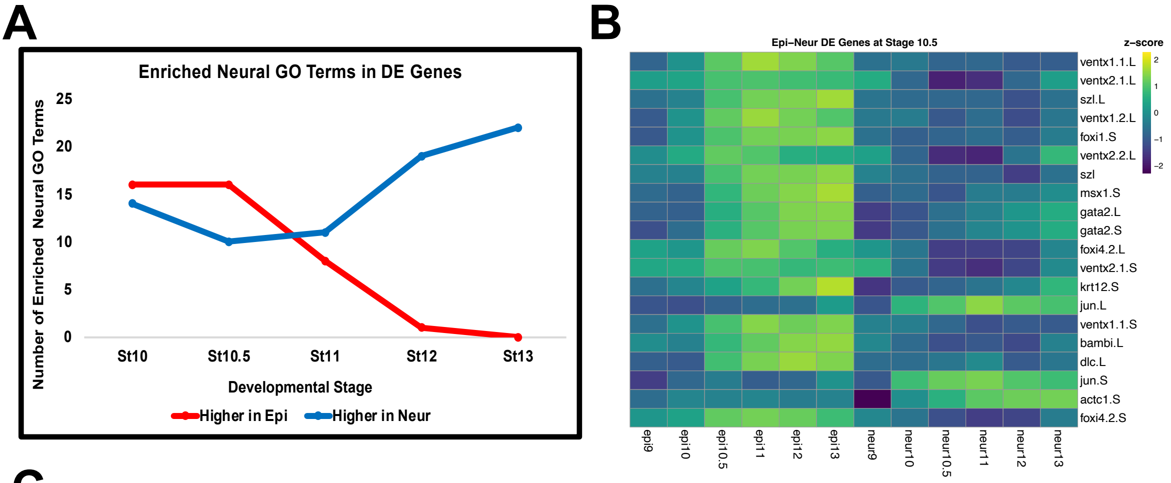

C

TGF-beta Kegg Enrichment in DE Genes					
**Stage**	**10**	**10.5**	**11**	**12**	**13**
**Epi**		+	+	+	+
**Neur**					

Supplemental Figure 4. Epidermal and Neural Lineages Diverge at St 10.5 (A) Number of enriched Neural GO Terms in genes significantly higher in the neural lineage (blue) and epidermal lineage (red) at each developmental stage. (B) Heatmap of the top 20 DE genes by Log2FC with a minimum expression of 10TPM between the epidermal and neural lineages at stage 10.5 (C) KEGG enrichment analysis of genes differentially expressed between epidermal and neural lineage at each developmental stage. Genes significantly increased in the epidermal lineage are enriched for TGF-beta genes, as defined by KEGG database from stages 10.5-13.
