# Supplementary material for: Quantitative analysis of transcriptome dynamics provides novel insights into developmental state transitions
Source: BMC Genomics. 2022 Oct 23;23:723. doi: 10.1186/s12864-022-08953-3 (PMC9588240; doi:10.1186/s12864-022-08953-3)
Supplement: Supplementary file 5 — Additional file 5: Supplemental Figure 5. BMP Ventralizes Mesoderm. (A)Western blot analysis of lysates of developing mesoderm (20ng/uL BMP4/7) and endoderm (160ng/uL Activin) explants for pSmad2 and Smad2 with Actin loading control. Gels were cropped to minimize empty space, uncropped gels are Supplemental Figure 9A. (B) PCA of published Activin induced mesoderm data (Satou-Kobayashi et al. 2021) with our ventral mesoderm and endoderm data shows that activin induced mesoderm clusters with our activin induced endoderm rather than BMP4/7 induced mesoderm. (C) Hierarchical clustering of Dorsal/Ventral genes (Ding et al. 2017) shows that endoderm and ventral mesoderm cluster on opposite sides of the heat map, suggesting BMP4/7 effectively ventralizes the mesoderm. [file 12864_2022_8953_MOESM5_ESM.pdf]

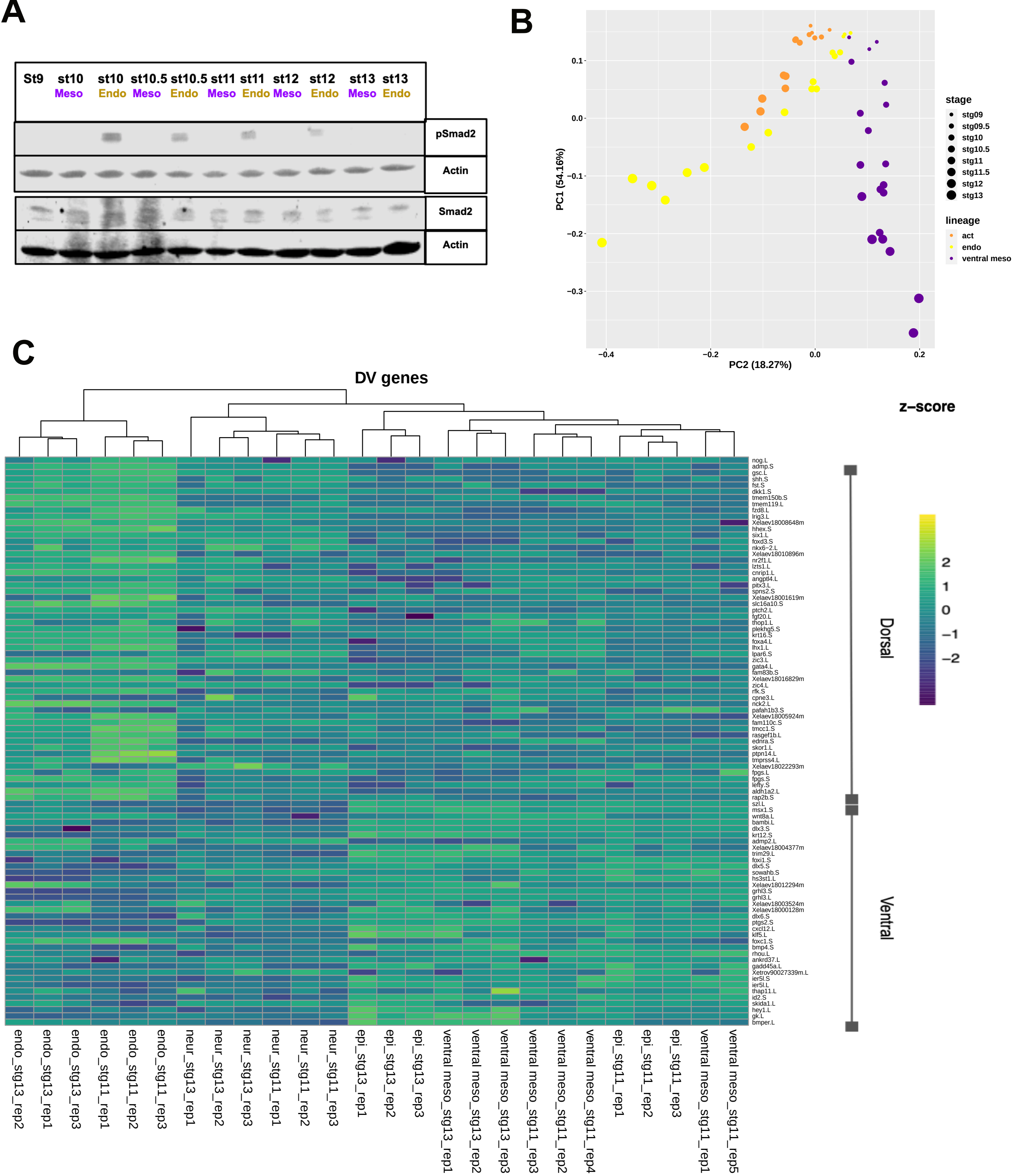

Supplemental Figure 5. BMP Ventralizes Mesoderm. (A)Western blot analysis of lysates of developing mesoderm (20ng/uL BMP4/7) and endoderm (160ng/uL Activin) explants for pSmad2 and Smad2 with Actin loading control. Gels were cropped to minimize empty space, uncropped gels are Supplemental Figure 9A. (B) PCA of published Activin induced mesoderm data (Satou-Kobayashi et al. 2021) with our ventral mesoderm and endoderm data shows that activin induced mesoderm clusters with our activin induced endoderm rather than BMP4/7 induced mesoderm. (C) Hierarchical clustering of Dorsal/Ventral genes (Ding et al. 2017) shows that endoderm and ventral mesoderm cluster on opposite sides of the heat map, suggesting BMP4/7 effectively ventralizes the mesoderm.
