# Supplementary material for: Quantitative analysis of transcriptome dynamics provides novel insights into developmental state transitions
Source: BMC Genomics. 2022 Oct 23;23:723. doi: 10.1186/s12864-022-08953-3 (PMC9588240; doi:10.1186/s12864-022-08953-3)
Supplement: Supplementary file 6 — Additional file 6: Supplemental Figure 6. Mesendoderm Analysis. (A) Number of differentially expressed genes between the endoderm and ventral mesoderm lineages at each developmental stage (padj ≤ 0.05). (B) KEGG enrichment analysis of genes differentially expressed between ventral mesoderm and endoderm lineage at each developmental stage. Genes significantly increased in the endoderm lineage are enriched for TGF-beta genes, as defined by KEGG database from stages 10-10.5 and genes significantly higher in the ventral mesoderm lineage are enriched for TGFbeta genes for stages 11-12. (C) WGCNA on stages 10,10.5 and 11 in the mesoderm and endoderm lineages identifies 22 gene modules, the blue module demonstrating increasing correlation over time to the endoderm lineage and the brown module demonstrating increasing correlation over time to the mesoderm lineage. [file 12864_2022_8953_MOESM6_ESM.pdf]

A

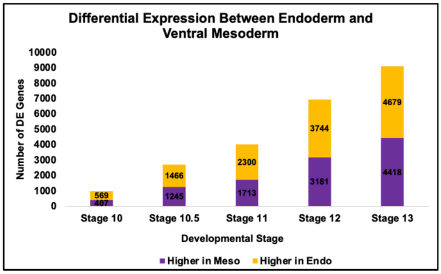

B

TGF-beta Kegg Enrichment in DE Genes:					
Stage	10	10.5	11	12	13
Endo	+	+			
Meso			+	+	

C

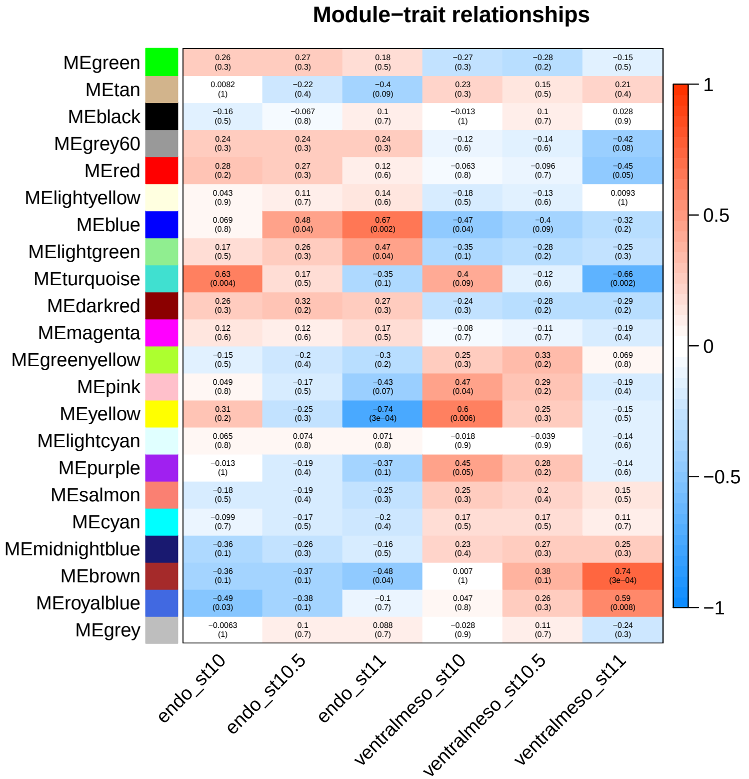

Supplemental Figure 6. Mesendoderm Analysis. (A) Number of differentially expressed genes between the endoderm and ventral mesoderm lineages at each developmental stage ($p_{adj} \leq 0.05$). (B) KEGG enrichment analysis of genes differentially expressed between ventral mesoderm and endoderm lineage at each developmental stage. Genes significantly increased in the endoderm lineage are enriched for TGF-beta genes, as defined by KEGG database from stages 10-10.5 and genes significantly higher in the ventral mesoderm lineage are enriched for TGF-beta genes for stages 11-12. (C) WGCNA on stages 10,10.5 and 11 in the mesoderm and endoderm lineages identifies 22 gene modules, the blue module demonstrating increasing correlation over time to the endoderm lineage and the brown module demonstrating increasing correlation over time to the mesoderm lineage.
