# Supplementary material for: Quantitative analysis of transcriptome dynamics provides novel insights into developmental state transitions
Source: BMC Genomics. 2022 Oct 23;23:723. doi: 10.1186/s12864-022-08953-3 (PMC9588240; doi:10.1186/s12864-022-08953-3)
Supplement: Supplementary file 7 — Additional file 7: Supplemental Figure 7. Early not Exogenous BMP Causes Mesoderm Formation. (A) Number of differentially expressed genes between the epidermal and ventral mesoderm lineages at each developmental stage (padj ≤ 0.05). (B) Number of enriched Mesoderm GO Terms (solid line) and Epidermis GO Terms (dashed line) in genes significantly higher in the ventral mesoderm lineage (purple) and epidermal lineage (red) at each developmental stage. (C) Western Blot Analysis of lysates for epidermal (WT) and BMP4/7 treated at stage 9 (BMP4/7 20ng/uL) explants collected at stage 10 and epidermal (WT) and BMP4/7 treated at stage 10.5 (BMP4/7 20ng/uL) explants collected at stage 11 for pSmad1/5/8 with Actin loading control. Gel was cropped to show one replicate, uncropped gel is Supplemental Figure 9B. (D) qRT-PCR of animal pole explants examining the fold change from stage 9 to 13 of expression of mesodermal marker Bra(T) for epidermis(WT), treated with BMP4/7(20ng/uL) at stage 9 and treated with BMP4/7 (20ng/uL) at stage 10.5 (***P<0.005). [file 12864_2022_8953_MOESM7_ESM.pdf]

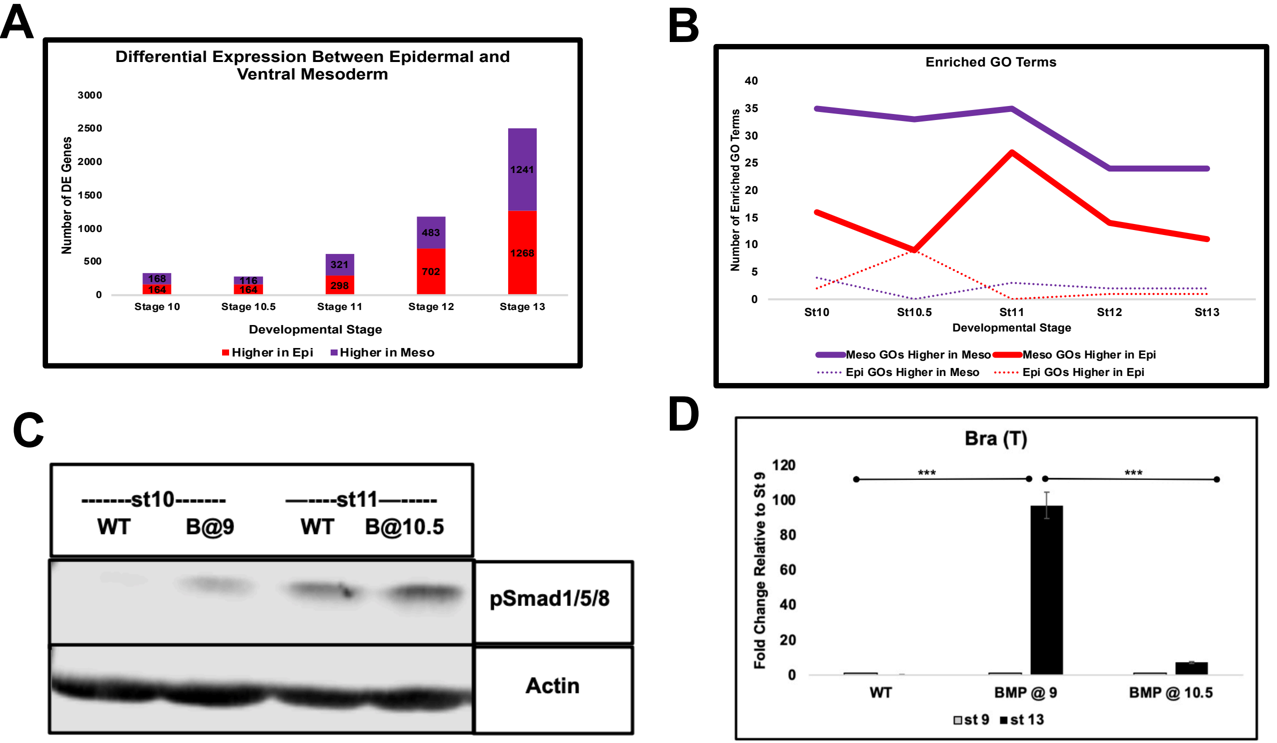

Supplemental Figure 7 Early not Exogenous BMP Causes Mesoderm Formation. (A) Number of differentially expressed genes between the epidermal and ventral mesoderm lineages at each developmental stage ($p_{adj} \leq 0.05$). (B) Number of enriched Mesoderm GO Terms (solid line) and Epidermis GO Terms (dashed line) in genes significantly higher in the ventral mesoderm lineage (purple) and epidermal lineage (red) at each developmental stage. (C) Western Blot Analysis of lysates for epidermal (WT) and BMP4/7 treated at stage 9 (BMP4/7 20ng/uL) explants collected at stage 10 and epidermal (WT) and BMP4/7 treated at stage 10.5 (BMP4/7 20ng/uL) explants collected at stage 11 for pSmad1/5/8 with Actin loading control. Gel was cropped to show one replicate, uncropped gel is Supplemental Figure 9B. (D) qRT-PCR of animal pole explants examining the fold change from stage 9 to 13 of expression of mesodermal marker *Bra(T)* for epidermis(WT), treated with BMP4/7(20ng/uL) at stage 9 and treated with BMP4/7 (20ng/uL) at stage 10.5 (****P*<0.005).
