# Supplementary material for: Quantitative analysis of transcriptome dynamics provides novel insights into developmental state transitions
Source: BMC Genomics. 2022 Oct 23;23:723. doi: 10.1186/s12864-022-08953-3 (PMC9588240; doi:10.1186/s12864-022-08953-3)
Supplement: Supplementary file 8 — Additional file 8: Supplemental Figure 8. Raw Images of Main Figure Western Blots. (A) Figure 4A Western blot analysis of lysates of developing epidermal (WT) and neural (20uM K02288) explants for pSmad1/5/8 and Smad1 with Actin loading control. Blot was cut prior to antibody hybridization to conserve antibody, unedited scan showing blot edges provided. (B) Figure 7A Western Blot analysis of lysates for developing epidermal (WT) and ventral mesoderm (BMP4/7 20ng/uL) explants for pSmad1/5/8 and Smad1 with Actin loading control. Blot was cut prior to antibody hybridization to conserve antibody. Initial scan showing blot edges (top) and high resolution unedited scan of relevant size (bottom) both provided. (C) Figure 8C Western Blot of lysates for stage 10 epidermal (WT), Dand5 MO injected (40pmol/embryo), ventral mesoderm (BMP4/7 20ng/uL), and endoderm (160ng/uL Activin) explants for pSmad1/5/8 with Actin loading control. Blot was cut prior to antibody hybridization to conserve antibody. Initial scans showing blot edges and high resolution unedited scans of relevant size for all three replicates are provided. Figure 8C is replicate 1. [file 12864_2022_8953_MOESM8_ESM.pdf]

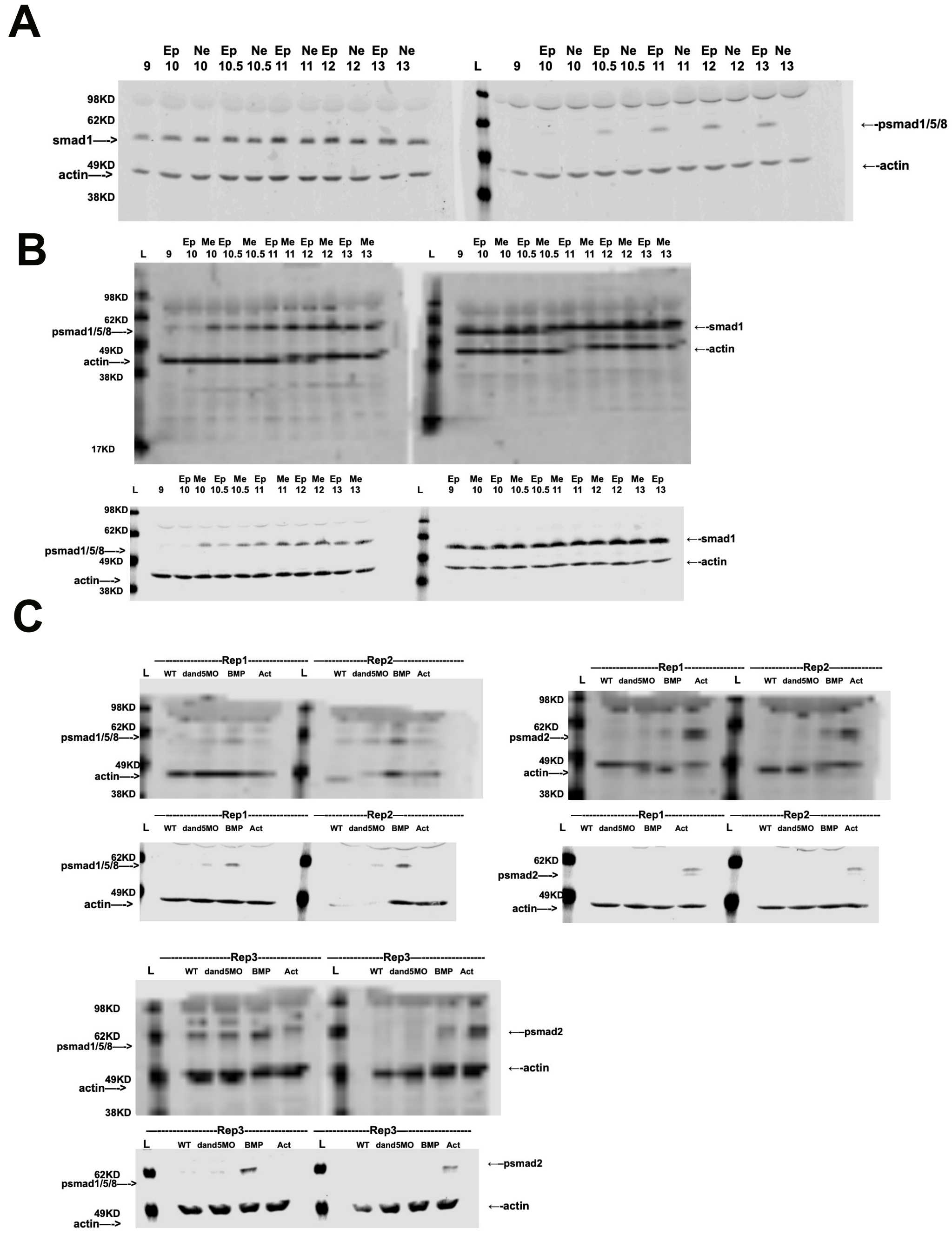

Supplemental Figure 8. Raw Images of Main Figure Western Blots. (A) Figure 4A Western blot analysis of lysates of developing epidermal (WT) and neural (20uM K02288) explants for pSmad1/5/8 and Smad1 with Actin loading control. Blot was cut prior to antibody hybridization to conserve antibody, unedited scan showing blot edges provided. (B) Figure 7A Western Blot analysis of lysates for developing epidermal (WT) and ventral mesoderm (BMP4/7 20ng/uL) explants for pSmad1/5/8 and Smad1 with Actin loading control. Blot was cut prior to antibody hybridization to conserve antibody. Initial scan showing blot edges (top) and high resolution unedited scan of relevant size (bottom) both provided. (C) Figure 8C Western Blot of lysates for stage 10 epidermal (WT), *Dand5* MO injected (40pmol/embryo), ventral mesoderm (BMP4/7 20ng/uL), and endoderm (160ng/uL Activin) explants for pSmad1/5/8 with Actin loading control. Blot was cut prior to antibody hybridization to conserve antibody. Initial scans showing blot edges and high resolution unedited scans of relevant size for all three replicates are provided. Figure 8C is replicate 1.
