# Supplementary material for: Quantitative analysis of transcriptome dynamics provides novel insights into developmental state transitions
Source: BMC Genomics. 2022 Oct 23;23:723. doi: 10.1186/s12864-022-08953-3 (PMC9588240; doi:10.1186/s12864-022-08953-3)
Supplement: Supplementary file 9 — Additional file 9: Supplemental Figure 9. Raw Images of Supplemental Western Blots. (A) Supplemental Figure 5A Western blot analysis of lysates of developing mesoderm (20ng/uL BMP4/7) and endoderm (160ng/uL Activin) explants for pSmad2 and Smad2 with Actin loading control. Blot was cut prior to antibody hybridization to conserve antibody. Initial scan showing blot edges (top) and unedited high resolution scan of relevant size bands (bottom) both provided. (B) Supplemental Figure 7C Western Blot Analysis of lysates for epidermal (WT) and BMP4/7 treated at stage 9 (BMP4/7 20ng/uL) explants collected at stage 10 and epidermal (WT) and BMP4/7 treated at stage 10.5 (BMP4/7 20ng/uL) explants collected at stage 11 for pSmad1/5/8 with Actin loading control. Blot was cut prior to hybridization with antibody to conserve antibody. Initial scan showing blot edges (top) and unedited high resolution scan of revelant size bands (bottom) both provided. All three replicates are shown, Supplemental Figure 6A is replicate 1. [file 12864_2022_8953_MOESM9_ESM.pdf]

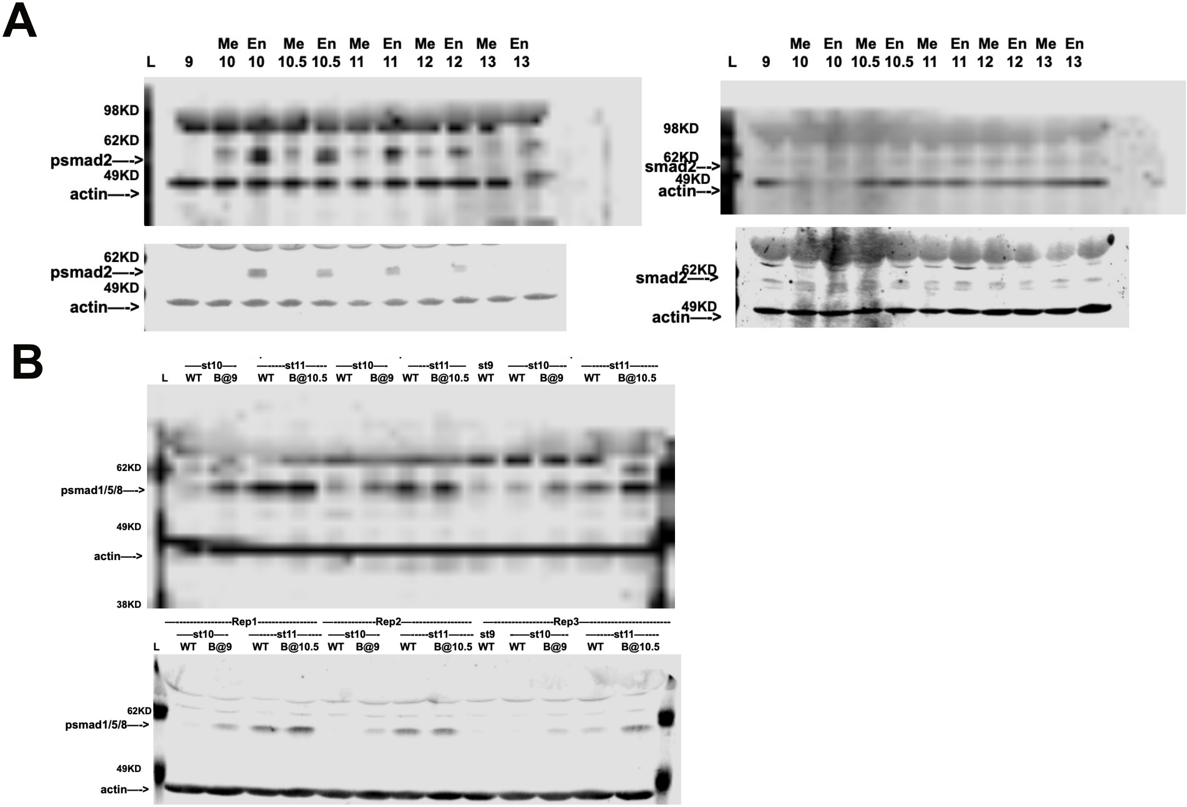

Supplemental Figure 9. Raw Images of Supplemental Western Blots. (A) Supplemental Figure 5A Western blot analysis of lysates of developing mesoderm (20ng/uL BMP4/7) and endoderm (160ng/uL Activin) explants for pSmad2 and Smad2 with Actin loading control. Blot was cut prior to antibody hybridization to conserve antibody. Initial scan showing blot edges (top) and unedited high resolution scan of relevant size bands (bottom) both provided. (B) Supplemental Figure 7C Western Blot Analysis of lysates for epidermal (WT) and BMP4/7 treated at stage 9 (BMP4/7 20ng/uL) explants collected at stage 10 and epidermal (WT) and BMP4/7 treated at stage 10.5 (BMP4/7 20ng/uL) explants collected at stage 11 for pSmad1/5/8 with Actin loading control. Blot was cut prior to hybridization with antibody to conserve antibody. Initial scan showing blot edges (top) and unedited high resolution scan of revelant size bands (bottom) both provided. All three replicates are shown, Supplemental Figure 6A is Rep 1.
